# Efficacy of i-Scan Imaging for the Detection and Diagnosis of Early Gastric Carcinomas

**DOI:** 10.1155/2014/819395

**Published:** 2014-03-09

**Authors:** Junichi Nishimura, Jun Nishikawa, Munetaka Nakamura, Atsushi Goto, Kouichi Hamabe, Shinichi Hashimoto, Takeshi Okamoto, Masato Suenaga, Yusuke Fujita, Yoshihiko Hamamoto, Isao Sakaida

**Affiliations:** ^1^Department of Gastroenterology and Hepatology, Yamaguchi University Graduate School of Medicine, 1-1-1 Minami-kogushi, Ube, Yamaguchi 755-8505, Japan; ^2^Department of Biomolecular Engineering Applied Molecular Bioscience, Yamaguchi University Graduate School of Medicine, Ube, Yamaguchi 755-8611, Japan

## Abstract

We determined comparative efficacy of i-Scan for detection and diagnosis of gastric cancer. Ten patients diagnosed with early gastric cancer based on histopathological findings were analyzed. White light and i-Scan moving images recorded from these patients in twin mode were separated into white light and i-Scan. Twelve endoscopists (three different skill levels) blinded to patient information evaluated the images. Correlation between demarcation accuracy and lesion brightness on still images was investigated. No significant differences were found in diagnostic accuracy between white light and i-Scan moving images for tumor detection rate (91.7% versus 90.8%, *P* = 0.777). Diagnostic accuracy of tumor size was comparable between novice and experienced endoscopists for i-Scan moving images (65.7% versus 71.1%, *P* = 0.528), whereas it was significantly lower for white light moving images (41.2% versus 79.5%, *P* = 0.019). Tumor demarcation accuracy was significantly better with white light than i-Scan still images (71.0% versus 65.8%, *P* = 0.033). Correlations between demarcation accuracy and brightness reached highs of 0.75 for white light and 0.89 for i-Scan imaging. Efficacy of i-Scan over that of white light imaging for detecting and diagnosing gastric cancer was not shown; however, the diagnostic capability of i-Scan can be improved if imaging conditions are optimized.

## 1. Introduction

Esophagogastroduodenoscopy (EGD) is widely used for the screening of gastric cancer in Japan. Detection of early gastric cancers is difficult because a diagnosis must be made based on minute irregularities and subtle changes in color of the mucosal surface. Such cancers are often missed by EGD, and the reported miss rate for gastric cancers is approximately 20% [1]. Image-enhanced endoscopy (IEE) has advanced greatly in recent years [2], and narrow-band imaging (NBI) is reported to be useful for the diagnosis of differentiation and demarcation of gastric tumors owing to enhanced visualization of the mucosal structure and microvessels [3, 4]. Flexible spectral imaging color enhancement (FICE) is another type of IEE based on spectral image processing technology. The FICE system provides high-contrast images by enhancement of differences in color between the tumor and normal mucosa [5, 6]. However, these techniques have not been sufficient to improve the detection rate of gastric cancers.

Among the image-enhancing techniques, i-Scan is a new computerized dynamic digital image processor that provides enhanced high-resolution images. i-Scan combines high-resolution endoscopy with 3 adjustable modes of image enhancement: surface enhancement (SE), contrast enhancement (CE), and tone enhancement (TE). SE enhances light-dark contrast, and CE digitally adds blue color to relatively dark areas by obtaining luminance intensity data for each pixel. Applying SE and CE allows for detailed observation of subtle irregularities around the tissue surface without reducing the light source. TE analyzes the individual red, green, and blue components of a normal image and recombines the color frequencies of each component to enhance minute mucosal structures with subtle color changes. The TE mode is similar to NBI and FICE and could be suitable for the qualitative diagnosis of a detected lesion [7]. The efficacy of i-Scan in the detection and histological prediction of colorectal cancer in colonoscopy has been shown in [8, 9], but its efficacy for gastric cancer has not been reported.

In this study, we aimed to determine the efficacy of i-Scan for detecting gastric cancer by evaluating separate white light (WL) and i-Scan images that were originally recorded simultaneously in twin mode.

## 2. Materials and Methods

### 2.1. Subjects

Subjects were 10 patients (10 lesions) who underwent EGD at Yamaguchi University Hospital between July and September 2010 and were subsequently diagnosed with early gastric cancer based on the histopathological findings from resected specimens. Clinicopathological features are shown in Table 1. Endoscopic submucosal dissection was performed in 7 and surgical resection in 3 patients, and 7 well-differentiated and 3 poorly differentiated tumors were found. With regard to the depth of tumor invasion, 1 patient had submucosal cancer and the other 9 patients had mucosal cancer. After receiving explanations of the purpose of and procedure involved in this study, all patients provided their written informed consent to undergo EGD prior to treatment.

### 2.2. i-Scan and Twin Mode

**Figure 1:**
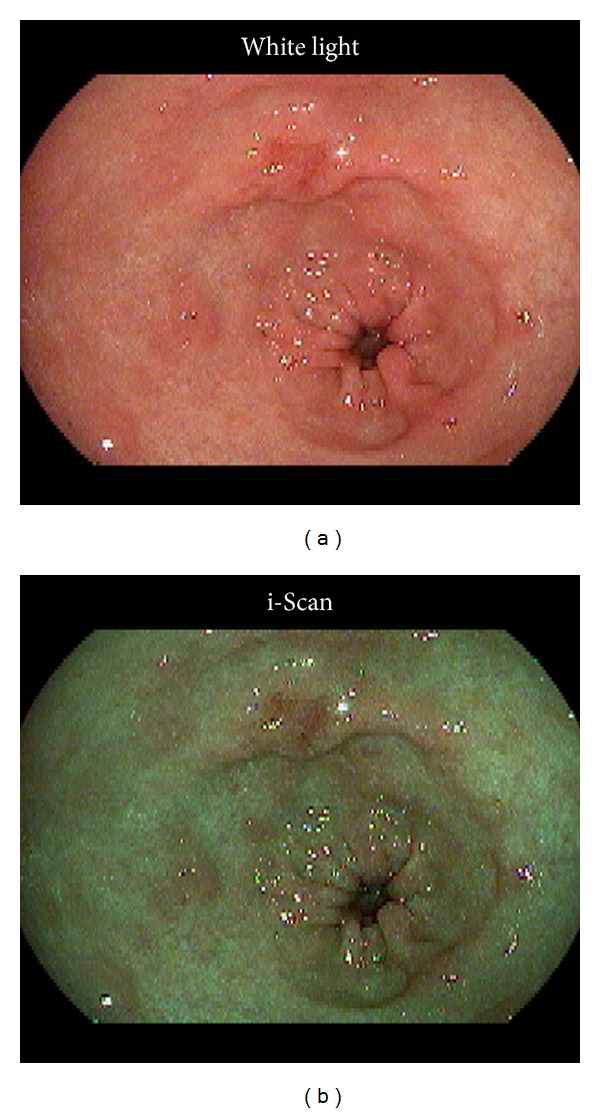
A still image of an early gastric cancer type 0-IIc in the lesser curvature of the gastric antrum. White light (a) and i-Scan (b) images were displayed in twin mode.

EGD was performed using a PENTAX EG29-i10N endoscope (HOYA, Tokyo, Japan) and an EPK-I processor (HOYA). We used the i-Scan TE-g mode, which was developed specifically to examine gastric lesions and to enhance color differences between normal mucosa and neoplastic lesions [7]. WL and i-Scan moving images were recorded continuously in twin mode from the cardia to pyloric ring (Video 1 in Supplementary Material available online at http://dx.doi.org/10.1155/2014/819395) in forward and reverse directions. The recording of the moving images was done in analog with a DVD recorder, and then the images were converted to mpeg files. Twin-mode moving images and the representative still images of the lesions (Figure 1) were then separated into respective WL and i-Scan images for evaluation.

### 2.3. Evaluators

The endoscopic images were evaluated by 12 gastrointestinal endoscopists blinded to patient information: 4 novice endoscopists with experience of <500 EGD cases, 4 intermediate endoscopists with experience of 500–3000 cases, and 4 advanced endoscopists with experience of >3000 cases who were also specialists certified by the Japan Gastroenterological Endoscopy Society. One of the novice endoscopists and 2 of the intermediate endoscopists had previously used i-Scan <50 times.

### 2.4. Assessment of Gastric Cancer Detectability and Diagnosis

Moving images from the 10 patients were divided into WL and i-Scan images, and 20 image files were prepared for evaluation (Video 1 in Supplementary Material available online at http://dx.doi.org/10.1155/2014/819395). Evaluators were informed that one pathologically defined gastric cancer lesion was present in each moving image. In the initial assessment, they examined WL moving images from 5 patients and i-Scan moving images from the remaining 5 patients. Moving images were played by Windows Media Player. Evaluators were allowed to pause, replay, forward, and rewind the moving images for 10 min per image file. During the evaluation, they determined the tumor location, size, invasion depth, and macroscopic type. The stomach was anatomically divided into the cardia, fundus, body, and pylorus, and the cross-sectional circumference was divided into the anterior and posterior walls and the greater and lesser curvatures. Tumors were classified according to diameter (≤10, 11–20, 21–30, or ≥31 mm) and macroscopic type (0-I, 0-IIa, 0-IIb, 0-IIc, or 0-III). Depth of tumor invasion was recorded as mucosal (m) or submucosal (s) or involving the muscularis propria (mp). Classification of gastric cancer was performed in accordance with the Japanese Classification of Gastric Carcinoma (3rd edition) [10]. Three months after the initial assessment, when memory of the initial assessment would not affect a second assessment, the evaluators evaluated the remaining 5 WL and 5 i-Scan moving images they had not yet seen.

Tumor detection was considered as correct when the evaluators accurately determined the gastric region and circumference of the tumors. If the evaluators could not determine tumor location correctly, subsequent answers provided about tumor size, macroscopic type, and invasion depth were excluded from the assessment. Data from the initial and second assessments were combined and analyzed to determine the efficacy of i-Scan for the detection and diagnosis of gastric carcinomas. We also investigated the effect of different levels of experience on diagnostic accuracy when using the WL and i-Scan methods.

### 2.5. Assessment of Demarcation Accuracy

**Figure 2:**
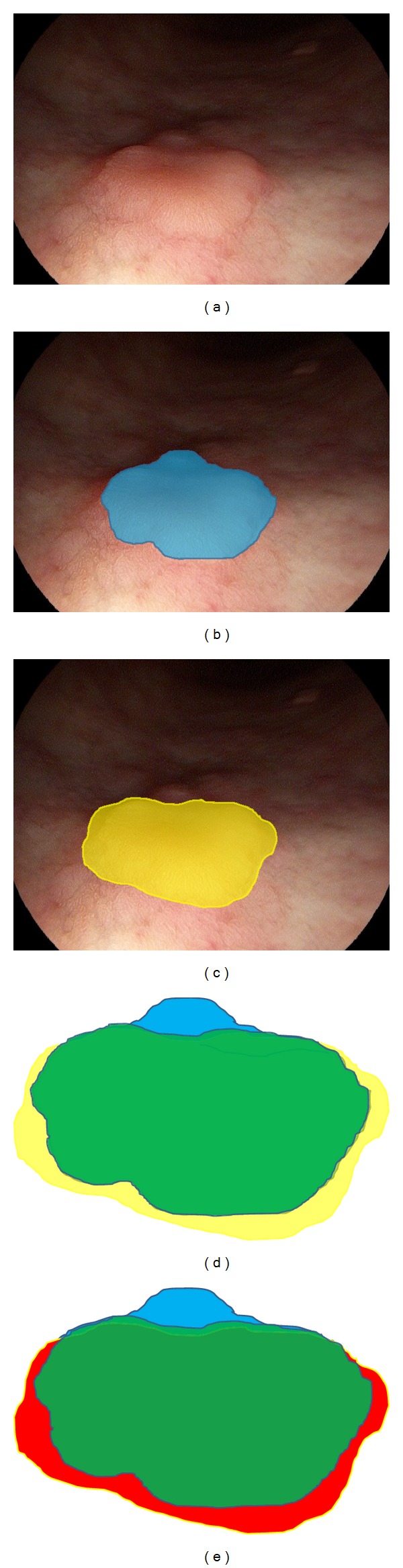
Assessment of demarcation accuracy. Endoscopic image of type 0-IIa gastric cancer in the greater curvature of the lower body.

Representative still images from the 10 tumors were divided into WL and i-Scan images, and 20 still image files were prepared for evaluation (Figure 1). Following the evaluation of the moving images, the evaluators were presented with WL still images for 5 tumors and i-Scan still images for the remaining 5 tumors that were matched with the moving images of the initial assessment. Ten still images printed in color were prepared to mark the tumor margins. Three months later, the evaluators demarcated the tumor on the remaining 5 WL and 5 i-Scan still images. Demarcation accuracy by individual evaluators was compared with histopathologically correct demarcation agreed upon by two specialists (JN, TO) certified by the Japan Gastroenterological Endoscopy Society. Images with demarcation lines drawn by the individual evaluators and by the specialists were scanned and superimposed on a computer screen to measure the percentage of areas of correct and incorrect tumor demarcation as determined by pixel counts (Figure 2). Endoscopic still image of type 0-IIa gastric cancer is shown in Figure 2(a). The area of correct demarcation based on the histopathological evaluation of the resected specimen is shown in blue, Figure 2(b), the area of demarcation determined by an individual endoscopist in yellow, Figure 2(c), and the correct area of demarcation achieved by the endoscopist in green, Figure 2(d)). The percentage of areas correctly demarcated is calculated by dividing the area in green by the area in blue. The area demarcated by the endoscopist (in yellow) not overlapping with the correct area of tumor (in blue), that is, the area without tumor, is shown in red (Figure 2(e)). The percent area incorrectly demarcated is calculated by dividing the area in red by the area in yellow. Accuracy of demarcation is calculated by subtracting red/yellow from green/blue. In this study, the difference between % correct and % incorrect was defined as the diagnostic accuracy of demarcation.

We measured brightness of tumorous areas determined by JN and TO on the same WL and i-Scan images by converting the color image into gray scale and subsequently calculating the mean gray-scale value of the pixels in the tumorous areas [11]. We investigated whether a correlation existed between diagnostic accuracy of demarcation and brightness for the tumorous areas.

### 2.6. Statistical Analysis

Statistical analysis was performed using the paired samples *t*-test, and regression analysis was used to determine the correlation between demarcation accuracy and lesion brightness. Significance was set at *P* < 0.05.

## 3. Results

**Figure 3:**
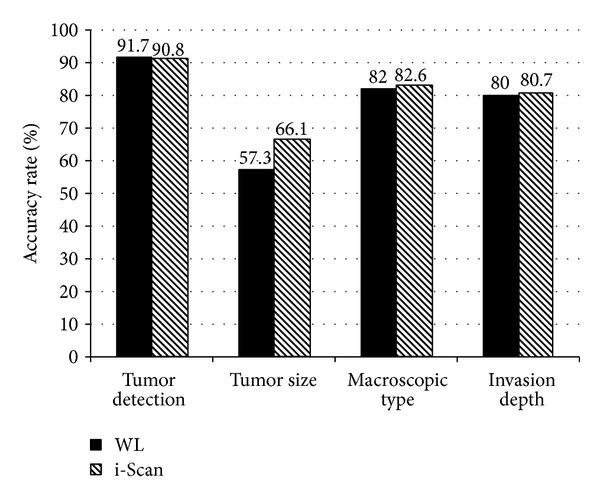
Outcome of tumor evaluation using white light (WL) and i-Scan moving images originally recorded in twin mode.

The tumor detection rate of gastric carcinomas from the moving images originally recorded in twin mode did not differ significantly between the WL and i-Scan images, at 91.7% (110/120) and 90.8% (109/120), respectively (*P* = 0.777). Diagnostic accuracy was not significantly different between WL and i-Scan images for any of the measurement parameters: tumor size, 57.3% (63/110) versus 66.1% (72/109; *P* = 0.173); macroscopic type, 82.0% (90/110) versus 82.6% (90/109; *P* = 0.988); and invasion depth, 80% (88/110) versus 80.7% (88/109; *P* = 0.684), respectively (Figure 3).

**Figure 4:**
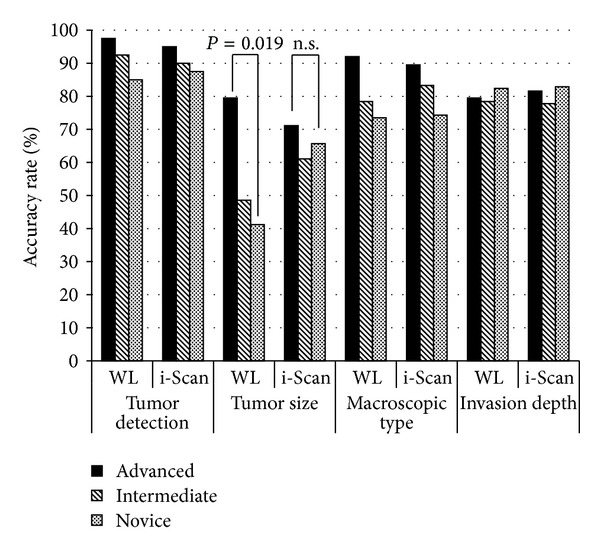
Difference by endoscopist skill level in tumor evaluation from white light (WL) and i-Scan moving images.

The diagnostic accuracy of tumor size when using i-Scan images was comparable between the novice and experienced endoscopists for i-Scan MI (65.7% versus 71.1%, *P* = 0.528) whereas it was significantly lower for WL MI (41.2% versus 79.5%, *P* = 0.019) (Figure 4). The accuracy of tumor size tended to be higher when using i-Scan versus WL images for intermediate endoscopists (48.6% versus 61.1%, *P* = 0.341) and novice endoscopists (41.2% versus 65.7%, *P* = 0.117). Overall, tumor detection, macroscopic size, and invasion depth did not differ significantly by experience level (Figure 4).

**Figure 5:**
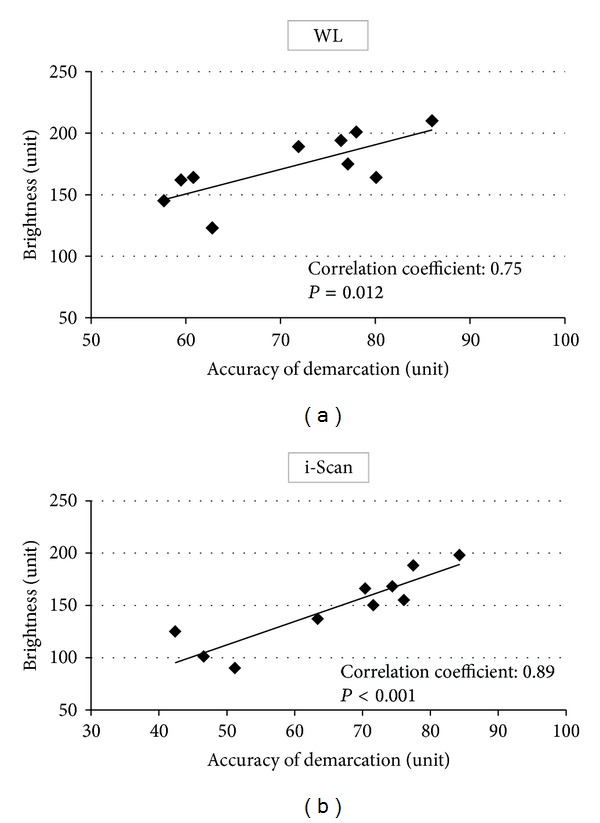
Correlation between accuracy of demarcation and lesion brightness. Correlation coefficients were 0.75 (*P* = 0.012) for white light (WL) (a) and 0.89 (*P* < 0.001) for i-Scan (b) imaging.

The accuracy of demarcation using still images was significantly higher with WL versus i-Scan images (71.0% versus 65.8%, *P* = 0.033). Mean brightness of the lesions was significantly lower on i-Scan images (147.8 units) than on WL images (172.7 units) (*P* < 0.001). An extremely high correlation between demarcation accuracy and lesion brightness was shown, with coefficients of 0.75 for WL images and 0.89 for i-Scan images (Figure 5).

## 4. Discussion

In the present study, i-Scan and WL images of early gastric carcinomas were recorded simultaneously in twin mode and then once separated, they were used to assess the accuracy of detection, diagnosis, and demarcation under the same condition in the same patient. Diagnostic accuracy when using moving images did not differ significantly between the two imaging methods. Although IEE is used to observe gastric carcinomas in detail, its efficacy in detecting gastric cancer has not yet been reported. Similarly, the present study showed no superiority of i-Scan over WL imaging. This is presumably because endoscopic diagnosis of gastric carcinomas is complicated by factors such as chronic gastritis in the background mucosa due to* H. pylori* infection, the fundic and pyloric glands having different ductal structures, and gastric cancer having varying degrees of tumor differentiation.

The results of tumor size measurement of novice endoscopists using i-Scan images were comparable with those of the experienced endoscopists. In a previous study where experts and nonexperts performed colonoscopy screening for colorectal carcinomas using WL and i-Scan images, it was found that although the tumor detection rate was similar between the two images for the experts, nonexperts had a significantly higher detection rate with i-Scan than with WL [12]. The diagnostic accuracy of novice endoscopists can be improved by using i-Scan images.

We clearly showed that the lesion brightness is low on i-Scan imaging, making the images darker, and an extremely high positive correlation existed between diagnostic accuracy of tumor demarcation and lesion brightness for both WL and i-Scan imaging. Although NBI has improved tumor detectability for organs with a narrow lumen, such as the esophagus and colon [13, 14], it is apparently not as effective for imaging of the wide lumen of the stomach due to insufficient brightness [15]. This study is the first, to our knowledge, to quantitatively show the importance of brightness in detecting tumors. When using i-Scan, it may be necessary to stay close to the tumor or increase the light intensity to obtain images with adequate brightness. We believe that the functional capabilities of i-Scan can be improved if imaging conditions, and particularly that of light intensity, are optimized for this modality.

Limitations of this study were its retrospective nature and small number of patients. In addition, because the i-Scan technology is relatively new, 3 of the 12 endoscopists had previously used it in <50 cases. More experience with i-Scan images may be necessary to spread this new technology more widely.

The diagnostic efficacy of i-Scan imaging over WL imaging for gastric cancer was not found in this study. The diagnostic accuracy of novice endoscopists can be improved by using i-Scan images. The functional capabilities of i-Scan can be improved if imaging conditions, and particularly that of light intensity, are optimized.

## Supplementary Material

A moving image of an early gastric cancer, type 0-IIc in the lesser curvature of the gastric antrum. White light (left) and i-Scan (right) images were displayed in twin mode.

## Figures and Tables

**Table 1:** Clinicopathological features of the early gastric carcinomas.

Macroscopic type	Color	Size (mm)	Differentiation	Invasion depth	ly	v	Therapy
0-IIc	Reddish	15	Differentiated	m	—	—	ESD
0-IIa	Reddish	8	Differentiated	m	—	—	ESD
0-IIc	Reddish	8	Differentiated	m	—	—	ESD
0-IIc	Reddish	13	Differentiated	m	—	—	ESD
0-IIc	Reddish	15	Undifferentiated	sm	—	—	Surgery
0-IIc	Discolored	29	Undifferentiated	m	—	—	Surgery
0-IIa	Normal-colored	12	Differentiated	m	—	—	ESD
0-IIc	Reddish	9	Differentiated	m	—	—	ESD
0-IIc	Discolored	25	Undifferentiated	m	—	—	Surgery
0-IIc	Reddish	20	Differentiated	m	—	—	ESD

m: mucosal cancer; sm: submucosal cancer; v: venous invasion; ly: lymphatic invasion; ESD: endoscopic submucosal dissection.

## References

[1] Aida K, Yoshikawa H, Mochizuki C (2008). Clinicopathological features of gastric cancer detected by endoscopy as part of annual health checkup. *Journal of Gastroenterology and Hepatology*.

[2] Takenaka R, Kawahara Y, Okada H (2009). Narrow-band imaging provides reliable screening for esophageal malignancy in patients with head and neck cancers. *The American Journal of Gastroenterology*.

[3] Yao K, Anagnostopoulos GK, Ragunath K (2009). Magnifying endoscopy for diagnosing and delineating early gastric cancer. *Endoscopy*.

[4] Ezoe Y, Muto M, Horimatsu T (2010). Magnifying narrow-band imaging versus magnifying white-light imaging for the differential diagnosis of gastric small depressive lesions: a prospective study. *Gastrointestinal Endoscopy*.

[5] Osawa H, Yoshizawa M, Yamamoto H (2008). Optimal band imaging system can facilitate detection of changes in depressed-type early gastric cancer. *Gastrointestinal Endoscopy*.

[6] Pohl J, May A, Rabenstein T, Pech O, Ell C (2007). Computed virtual chromoendoscopy: a new tool for enhancing tissue surface structures. *Endoscopy*.

[7] Kodashima S, Fujishiro M (2010). Novel image-enhanced endoscopy with i-scan technology. *World Journal of Gastroenterology*.

[8] Testoni PA, Notaristefano C, Vailati C, Di Leo M, Viale E (2012). High-definition colonoscopy with i-Scan: better diagnosis for small polyps and flat adenomas. *World Journal of Gastroenterology*.

[9] Lee CK, Lee S-H, Hwangbo Y (2011). Narrow-band imaging versus i-Scan for the real-time histological prediction of diminutive colonic polyps: a prospective comparative study by using the simple unified endoscopic classification. *Gastrointestinal Endoscopy*.

[10] Japanese Gastric Cancer Association (2011). Japanese classification of gastric carcinoma: 3rd English edition. *Gastric Cancer*.

[11] Jain AK (1989). *Fundamentals of Digital Image Processing*.

[12] Testoni PA, Notaristefano C, Di Leo M, Vailati C, Mazzoleni G, Viale E (2013). High-definition with i-Scan gives comparable accuracy for detecting colonic lesions by non-expert and expert endoscopists. *Digestive and Liver Disease*.

[13] Muto M, Minashi K, Yano T (2010). Early detection of superficial squamous cell carcinoma in the head and neck region and esophagus by narrow band imaging: a multicenter randomized controlled trial. *Journal of Clinical Oncology*.

[14] East JE, Suzuki N, Stavrinidis M, Guenther T, Thomas HJW, Saunders BP (2008). Narrow band imaging for colonoscopic surveillance in hereditary non-polyposis colorectal cancer. *Gut*.

[15] Kim KO, Ku YS (2013). Is image-enhanced endoscopy useful for the diagnosis and treatment of gastrointestinal tumor?. *Clinical Endoscopy*.

